# Hepatitis C Virus Clearance after Discontinuation of Pegylated Interferon Alpha-2a Monotherapy in a Child

**DOI:** 10.1155/2012/597348

**Published:** 2012-10-18

**Authors:** Takeshi Endo, Koichi Ito, Tokio Sugiura, Kenji Goto

**Affiliations:** Department of Pediatrics and Neonatology, Nagoya City University, Graduate School of Medical Sciences, Kawasumi, Nagoya 467-8601, Mizuho-Cho, Mizuho-Ku, Japan

## Abstract

The present patient was a 4-year-old boy. His hepatitis C virus genotype was 2a, and his viral load was high (1400,000 U/mL). The pretreatment liver biopsy revealed no fibrosis or malignancy and mild chronic hepatitis; his Knodell's histological activity (HAI) score was 4. Single nucleotide polymorphism of IL28B (rs8099917) was major type. The patient began antiviral treatment with pegylated interferon alpha 2a (90 **μ**g/week). At week 9, serum HCV RNA became undetectable, with a sensitivity of 50 copies/mL. Antiviral treatment was discontinued at week 11 because the ALT level increased to 610 U/L. After discontinuation of therapy, the patient's serum HCV RNA status became positive again. The serum viral load increased to 100,000 U/mL. During this period, he had been observed without medication. Sixteen months after stopping treatment, serum HCV became undetectable. Over a 4-year period, HCV RNA became negative and his anti-HCV antibody titer gradually decreased. In conclusion, though antiviral therapy resulted in failure or incomplete therapy, a reduced viral load resulted in viral clearance in the present patient. Interleukin 28B genotype might have association with the clearance of hepatitis C virus after discontinuation of antiviral therapy.

## 1. Introduction

About 70% of hepatitis C virus (HCV) infections lead to chronic liver disease, and chronic HCV infection leads to cirrhosis and hepatocellular carcinoma. Recently, antiviral therapy, such as pegylated interferon (PEG-IFN) and ribavirin, has been successful in more than 50% of infected patients, resulting in clearance of HCV RNA from the serum. It has been assumed that patients infrequently or never achieve elimination of chronic HCV infection without receiving antiviral treatment. Recent studies indicate that there is a relationship between rs8099917 TT interleukin (IL) 28B genotype and HCV treatment response in adults [1]. However, the IL28B genotype influences in HCV infection among children have been a little investigated. 

We report the case of a boy with viral clearance after discontinuation of PEG-IFN *α*2a monotherapy because of elevated transaminase levels. IL28 genotyping was performed in this patient. 

## 2. Case Presentation

**Figure 1:**
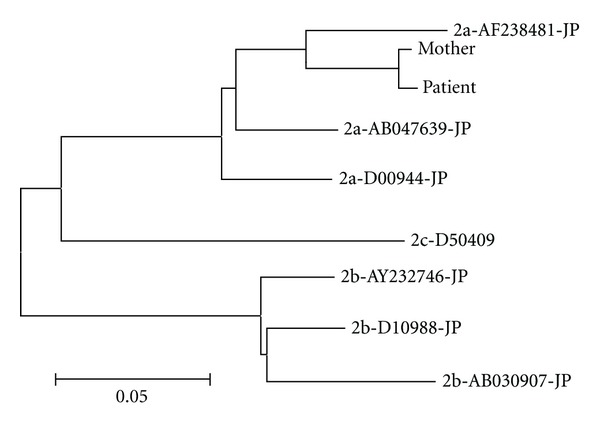
Phylogenetic analysis of hepatitis C virus in the patient and his mother. A phylogenetic tree was constructed using the neighbor-joining method. The horizontal axis shows the number of nucleotide substitutions per site. For example, 2a-AF238481-JP represents the strain of genotype 2a collected in Japan (registered no. AF238481 in GenBank).

A 2-year-old boy was referred to our hospital due to chronic hepatitis C infection. Markers of hepatitis A virus, hepatitis B virus, cytomegalovirus, and human immunodeficiency virus were negative. Tests for hepatitis E, and Epstein-Barr viruses were not performed. Autoantibody titers were not high, and serum IgG was within the normal range. The possibility of autoimmune hepatitis was excluded. In addition, the possibility of Wilson's disease was excluded because serum ceruloplasmin and copper levels were within the normal range. The patient had no history of herb consumption, use of holistic approach, blood transfusions, intravenous drug use, or tattooing. His mother had HCV antibodies and HCV RNA (genotype 2a). The patient-mother pair showed high similarity in the nonstructural 5B region (NS5B) on viral gene analysis (Figure 1). 

Therefore, this case was diagnosed as mother to child infection. The patient had been followed for over 2 years. He was asymptomatic and had no hepatosplenomegaly or jaundice. His height and body weight were appropriate for his age. 

At 4 years of age, because continuous transaminase elevation was observed, PEG-IFN monotherapy was started after obtaining written informed consent from his parents. When the antiviral therapy was started, his serum aspartate aminotransferase (AST) and alanine aminotransferase (ALT) levels were 41 U/L and 41 U/L, respectively (normal values ≤ 40 U/L); polymerase chain reaction blood analysis revealed HCV infection (genotype 2a; viral load 1400,000 U/mL). The pretreatment liver biopsy revealed no fatty liver, fibrosis or malignancy, and mild chronic hepatitis; his Knodell's histological activity (HAI) score was 4. Genotype of IL28B (single nucleotide polymorphism (SNP); rs8099917) was determined by polymerase chain reaction-direct sequencing method. SNPs of IL28B (rs8099917 TT) was major type. SNPs of IL28B (rs12979860) were not tested.

**Figure 2:**
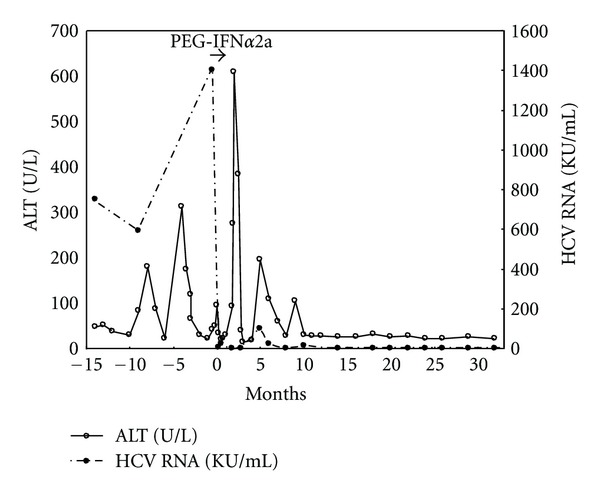
Biochemical and virological profile of the patients.

 The patient began antiviral treatment with PEG-IFN*α*2a (90 *μ*g/week subcutaneously); the drug dose was calculated based on his body surface area (BSA) according to the formula, BSA (m^2^)/(1.73 m^2^) × 180 *μ*g (adult dose). After antiviral therapy was started, the viral load decreased, with a rapid virologic response (viral load ≤ 5,000 U/mL) and with normalization of serum AST and ALT levels in about 1 month (Figure 2). At week 9, serum HCV RNA became undetectable, with a sensitivity of 50 copies/mL (Amplicore HCV v2.0 test; Roche Diagnostic). Antiviral treatment was discontinued at week 11 because the AST and ALT levels increased to 525 and 610 U/L, respectively. The patient had no symptom and showed normal coagulopathy. After discontinuation of therapy, the AST and ALT levels gradually decreased and normalized. Two months after stopping therapy, the patient's serum HCV RNA status became positive again, and his serum transaminase was mildly elevated. The serum HCV viral load increased to 100,000 U/mL, and the serum ALT increased to 195 U/L. During this period, he had been observed without medication. Four months after stopping treatment, his serum transaminase level and HCV viral load decreased gradually. Sixteen months after stopping treatment, serum HCV became undetectable. Over a 4-year period, HCV RNA became negative and his anti-HCV antibody titer gradually decreased. 

## 3. Discussion

In this case, HCV reappeared after discontinuation of antiviral therapy, and subsequently the patient achieved viral elimination without therapy. Spontaneous elimination of HCV is thought to be rare in individuals with persistent viral infection. Recently, two cohort studies reported that the incidence of spontaneous elimination of serum HCV RNA in chronic HCV carriers was 0.5%/year/person [2, 3]. This case is similar to the adult cases reported by Denis et al. [4]. They assumed that PEG-IFN plus ribavirin therapy was effective for the 2 patients, although the patients had previously had IFN monotherapy that resulted in failure. However, in the present case, the antiviral treatment was PEG-IFN monotherapy without ribavirin. The efficacy of PEG-IFN and ribavirin therapy has been reported in children. However, side effects of ribavirin, such as hemolytic anemia and teratogenicity, have been reported. Furthermore, the efficacy of PEG-IFN monotherapy has been reported in children [5]. In order to decrease the risk of side effects, the present patient was started on PEG-IFN monotherapy. 

In previously reported cases, spontaneous elimination of HCV occurred under particular circumstances, such as in younger children [6] and following delivery [7], immunosuppressive drugs [8], antiretroviral therapies against HIV [9], and surgical procedures [10]. In these reports, most of the cases achieved spontaneous elimination of HCV together with significant clinical events, which were typically associated with extraordinary changes in host immunity. In addition, several reports noted that spontaneous elimination of HCV was observed more often in patients with lower viral loads or lower levels of HCV core antigen [3, 6, 7]. Thus, it was assumed that, in the present case, pediatric immune status and lower viral load due to incomplete PEG-IFN therapy led to HCV elimination.

The interferon therapy is recommended in children three years age or older with the concerns of side effects such as spastic diplegia in reported young infants with hemangioma receiving interferon therapy. In addition, approximately 30% of infants who were HCV-infected through mother-to-infant transmission became negative for HCV-RNA in the blood during the natural course of infection by the time they were three years old [11]. Thus, this case was observed without antiviral treatment until four years of age. His viral load was very high, so that it was not considered that he would achieve spontaneous elimination without antiviral therapy. Antiviral therapy given at an early age is better tolerated than it is in adulthood; thus, early treatment is preferred to avoid the potential for serious liver disease. 

Recent study reported that IL 28B genotype was significantly associated with spontaneous clearance of HCV among infected children [12, 13]. That study focused on IL28 B (rs12979860 CC as a major type); however, IL 28B (rs12979860) was not tested in the present study. Meanwhile, the IL 28B genotype (rs8099917 TT) was tested and was found to be of the major (favorable) type [1]. In this patient IL 28B genotype (rs8099917) might have association with the clearance of HCV after discontinuation of antiviral therapy. Further large study to clarify IL 28B genotype and clearance of HCV in children is needed.

During antiviral therapy, a very high transaminase level was observed. Occasional patients experience an increase in serum transaminases during antiviral therapy, and this response has been little studied. It is assumed that hepatic iron overload or an accumulation of PEG-IFN is associated with transaminase elevation [14, 15]. However, in the present case, serum iron or ferritin was not measured during antiviral therapy, and liver biopsy was not performed after antiviral therapy. Thus, the cause of the elevated transaminase is unknown. Either way, we need to carefully consider whether or not to stop antiviral therapy immediately when patients develop increased serum transaminase levels. Stopping antiviral therapy temporarily and resuming it after normalization of transaminase levels may be worth considering. The patient had elevated transaminase during therapy could be an immuno modulating process which leads to viral clearance.

Recently, occult HCV infection has been described among HCV RNA-negative individuals in whom HCV RNA is detected in liver. Even in this case, the patient might have HCV in liver. Considering invasiveness of liver biopsy, liver biopsy has not done yet. But, long-term follow-up has been needed.

## 4. Conclusion

Though antiviral therapy resulted in failure or incomplete therapy, a reduced viral load resulted in viral clearance in the present patient. Interleukin 28B genotype might have association with the clearance of hepatitis C virus after discontinuation of antiviral therapy.

## Abbreviations

HCV: Hepatitis C virus
IL: Interleukin
PEG-IFN: Pegylated interferon
Ig: Immunoglobulin
AST: Aspartate aminotransferase
ALT: Alanine aminotransferase
SNP: Single nucleotide polymorphism.
